# Evaluation of environmental parameters in the Espinar Puno stabilization lagoon

**DOI:** 10.1016/j.heliyon.2021.e06959

**Published:** 2021-05-06

**Authors:** Lucio Ticona Carrizales, Polan Franbalt Ferró-Gonzales, Cynthia Milagros Apaza-Panca, Enrique Gualberto Parillo Sosa, Cristóbal Rufino Yapuchura Saico, Ingrid Rossana Rodríguez Chokewanca

**Affiliations:** a Facultad de Ingeniería Económica, Universidad Nacional del Altiplano, Puno, Perú; b Facultad de Administración Hotelera y de Turismo, Universidad Nacional de Frontera, Sullana, Piura, Perú; c Escuela Profesional de Gestión Pública y Desarrollo Social, Universidad Nacional de Juliaca, Juliaca, Perú

**Keywords:** Discharges, Inland Puno bay, Titicaca lake, Wastewater, Water pollution

## Abstract

The “Espinar” stabilization lagoon is a body of wastewater that is located in the district (region of Puno), which accumulates wastewater that comes from the population of the city of Puno-Peru. The present study was carried out in January and February of 2019 in affluent and effluent wastewater. The measures of pH, water temperature, total suspended solids, electrical conductivity and salinity were evaluated. The results showed that there are significant differences between the data of the affluent and effluent samples during the periods analyzed (Krustal Wallis). The median concentrations values of the effluent for the parameters of temperature (16.60 °C), salinity (0.67 mg/L), pH (7.70), total dissolved solids (669.00 ppm) and electrical conductivity (1463.07 μs/cm), they all show significant differences. Also, the removal efficiency was calculated by the total dissolved solids (TDS) and the positive removal of 7.80% of pollutant load was found. Although these results are within the established limits, the monitoring mechanisms must be established for an adequate control of the parameters evaluated and thus, there won't be a deterioration of the environment surrounding the lagoon.

## 1 Introduction

Lake Titicaca is of great importance for the city of Puno, where water is one of the most important source supply for the population. This problem undergoes an accelerated eutrophication process, due to poor wastewater management in the stabilization lagoon "El Espinar", by the service provider EMSAPUNO S.A. (Tudela, 2008).

The “Espinar” stabilization lagoon is structured in two lagoons arranged in series (primary and secondary), it began its operations in 1972, with the aim of treating the wastewater generated by the population of the city of Puno (TDPS-ALT, 2011). Although lagoons of this type have shown good performance for wastewater treatment with respect to biological oxygen demand and some pathogens, they face challenges of removal of suspended solids of phosphorus and total ammonia-nitrogen (Leblond, 2020).

When domestic wastewater are run off to water bodies without any treatment or disinfection, the contamination takes place with high concentrations of bacteria, viruses and parasites, creating a serious public health problem (Elías Silupu et al., 2020; Ferro et al., 2019; Ferro et al., 2019; Toledo Medrano and Amurrio Derpie, 2006).

According to Lampoglia (2001), potential of hydrogen (pH), is a parameter that affects the treatment processes, because the biological activity occurs satisfactorily in the pH range of 6.5–8. During the mornings the pH is slightly acidic, while in the afternoons is more alkaline. Temperature affects the physical, chemical and biological reactions that occur in a stabilization pond. The metabolism of microorganisms accelerates with increasing temperature, and slows down with its decrease. Generally, temperature also affects the concentration of dissolved oxygen. At high temperatures, the solubility of oxygen decreases and there is a tendency for its partial release to the atmosphere in situations of water supersaturation. Regarding the removal of total dissolved solids present in wastewater, it is one of the parameters to establish the efficiency of treatment systems. Electrical conductivity is an indication of the number of dissolved solids in wastewater. Likewise, it also mentions that industrial drains have high conductivity, due to the high concentration of dissolved solids.

In that same sense, Quezada Barahona and Rodríguez Rodríguez (2009) state that the appropriate pH value for different treatment processes and for the existence of biological life can be very restrictive and critical, however, it is generally 6.5–8.5. Likewise, Isasa (2000) indicates that the purpose of these limits is to avoid an undesirable transformation in the receiving body of the effluent both in the chemical aspects, with the probable formation of toxic or corrosive and microbiological compounds with the appearance of an inappropriate medium for the growth of the microbial flora.

Arias et al. (2001) found higher temperature values in summer and spring, while slightly lower values in autumn and winter in a study of Molle Bay. Isasa (2000) specifies that the temperature of the residual liquid is related to the concentration of dissolved oxygen that this water may have, since at higher temperatures it will contain less dissolved gases. Another consequence of the high temperature is the increase in the sedimentation rate of the materials in suspension, as the viscosity of the liquid mass decreases, which would cause the accumulation of sediments in places where this is not desirable.

Chalarca Rodríguez et al. (2007) affirm that there is an impact of wastewater on the swampy complex, however, these discharges mainly affect the environmental quality of the sites surrounding the urban area of the municipality of Ayapel. Ruiz Díaz et al. (2016), at the point of discharge of rain and sewage liquids in a part of the city of Corrientes, they observed a high value of 780 μs/cm.

Mejía Ruiz (2008) indicates that when there is no significant removal of contaminants, it could be an indication of a malfunction of the stabilization lagoon. For his part, Antonio (2000), mentions that in water, total solids produce light scattering when light passes through the body of water.

There are few studies that have correctly characterized the wastewater in order to calculate the input and output loads of the systems and find the efficiency in the removal of the parameters of interest. It is for this reason that load reductions in the presence of treatment systems must be estimated, but always indicating the elements that were taken into account to arrive at a given value (Pnuma et al., 1998).

MINAM (2010) through Supreme Decree No. 003-2010-MINAM, approves maximum permissible limits (MPL) for effluents from domestic or municipal wastewater treatment plants (see Table 1).

**Table 1:** Maximum permissible limits.

Parameter	Unit	MPL
Oils and fats	mg/l	20
Thermotolerant coliforms	NMP/ml	10000
DBO5	mg/l	100
Chemical oxygen demand	mg/l	200
pH	Unit	6.5–8.5
Total suspended solids	mg/l	150
Temperature	°C	<35

Therefore, the objectives of this study were: a) Analyze the variation in concentration of environmental parameters in the effluent wastewater and the effluent of the stabilization lagoon, by the parameter of: pH, water temperature, total suspended solids, electrical conductivity and salinity. b) Calculate the removal efficiency for the parameter of total dissolved solids, c) Compare with the Peruvian standard, on allowed values of concentration of discharges to the water body, of wastewater from the effluent of the stabilization lagoon “Espinar”.

## 2 Materials and methods

### 2.1 Study area

**Figure 1:**
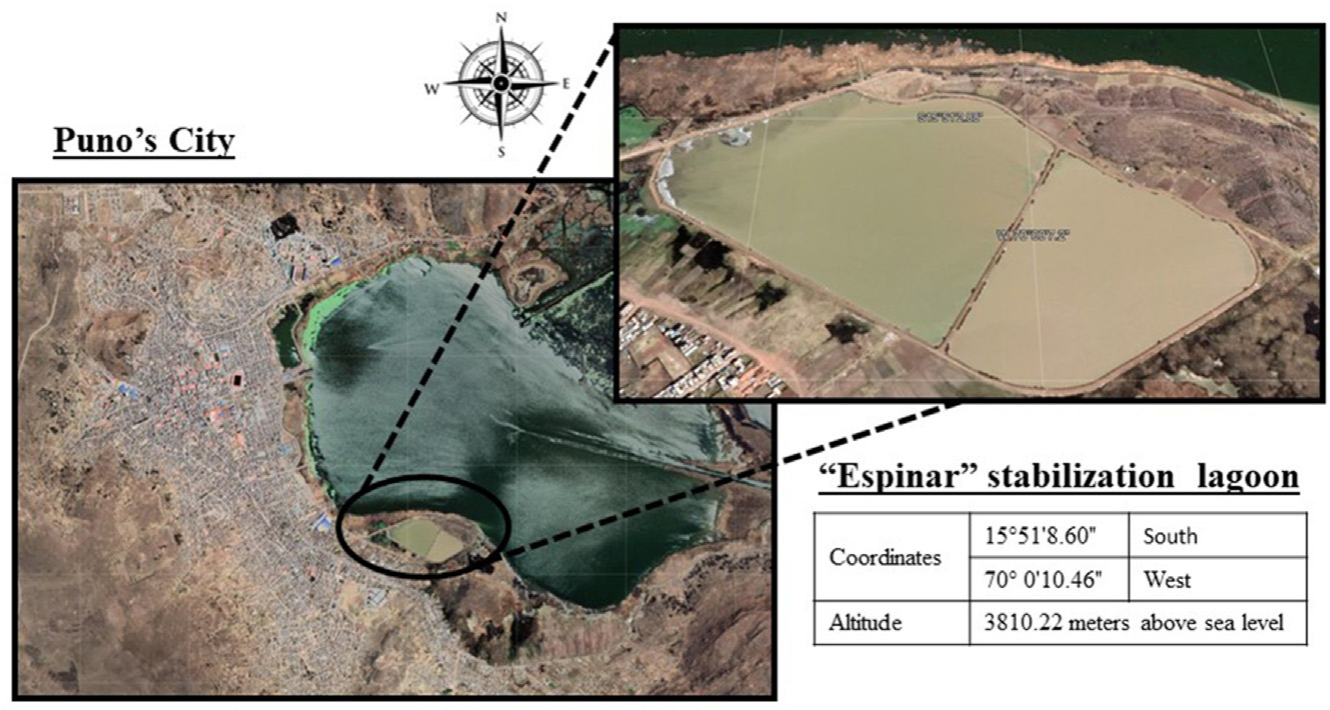
Study area.

Currently, the city of Puno has a stabilization lagoon "Espinar", which receives the drains collected from the main pumping stations that exist in the city (see Figure 1). This plant was built in flood-prone areas and is located in the extreme south of the city, bordering on the East with the population of the city of Puno, on the West with Espinar Island, on the North and South with the interior bay of Lake Titicaca. It is located at 3810.22 m above sea level, with the following coordinates: 15°51′8.60″S 70°0′10.46″W. The climate is temperate in the rainy summer, dry and cold in the autumn, winter and spring seasons.

### 2.2 Equipment used

For the present study, the HI 9828 “Hanna” multiparameter probe was used, which was introduced into the residual water of the affluent and effluent of the lagoon in a specific depth (4 m).

### 2.3 Data collection technique

We collected the effluent and affluent samples between 09:00 a.m. to 11:00 a.m. each 5 min, in a number of 10 repetitions in each place of reading data per day, respectively, on three different days (Monday, Wednesday and Friday) on February, getting a total of 30 samples.

Likewise, the HI 9828 “Hanna” multiparameter allowed us to work with its own specifications (see Table 2):

**Table 2:** Specifications of HI 9828 “Hanna” multiparametric meter probe.

Specifications	Parameters				
	Temperature	Salinity	pH	Total dissolved solids	Electric conductivity
Range	-5.00 to 55.00 °C	0.00 to 70.00 PSU	0.00–14.00	0–400000 mg/L	0.000–200.000 mS/cm
Accuracy	+/- 0.15 °C	0.10 PSU	+/- 0.02	+/- 1 mg/L	+/- 1 μs/cm

### 2.4 Evaluated parameters

In affluent wastewater and effluent from the lagoon, 5 environmental parameters were evaluated (pH, water temperature, total suspended solids, electrical conductivity and salinity).

Statistical analysis with Kruskal Wallis non-parametric ANOVA has been applied to compare two or more data populations. Of the evaluated parameters, whose distributions did not present a normal distribution and could not be mathematically normalized, the Kruskal Wallis statistical test was used, which allows a non-parametric analysis of variance, where those values of p < 0.05 indicate statistically significant differences (Balzarini et al., 2008).

#### 2.4.1 Total dissolved solids removal efficiency

In the present study, to calculate the percentage of hydraulic removal, we use the equation proposed by Romero-Aguilar et al. (2009).

Removal(%)=(Ci−Cf)∗100/Ci

Where:

*Ci* = Initial concentration of the parameter in the affluent.
*Cf* = Final concentration of the parameter in the effluent.

#### 2.4.2 Comparison of maximum permissible limits of the parameters

We made the comparison of the averages and medians of parameters of pH, temperature and total suspended solids, with the DS-003-2010-MINAN (MINAM, 2010).

## 3 Results and discussion

### 3.1 Analysis of the variation of concentration of the environmental parameters in the affluent wastewater and the effluent of the stabilization lagoon

#### 3.1.1 Temperature

The median value of temperature in the affluent (15.40 °C) with respect to the median value of the effluent (16.60 °C) show an increase (Table 3), and these differences are significant (Kruskal Wallis, p<0.05). This is possibly due to the fact that there is a gain of heat from the sun on the water surface of the secondary lagoon, in a rainy and temperate season. Likewise, Arias et al. (2001) in a study of Molle Bay, found in summer and spring, higher temperature values, while in autumn and winter, slightly lower values. A high temperature reported in the effluent is beneficial for the sedimentation of suspended matter in the stabilization lagoon, as indicated by Isasa (2000).

**Table 3:** Characteristics of the affluent wastewater to the Espinar lagoon and effluent into the Titicaca lake.

Parameter	Temperature °C		Salinity Milligram per Lite		pH value		TDS Parts per million		Electric conductivity microSiemens per centimeter	
	In	Out	In	Out	In	Out	In	Out	In	Out
Max.	15.50	17.80	0.97	0.88	7.93	7.73	943.00	863.00	1884.00	1726.00
Min.	15.20	15.90	0.72	0.63	7.67	7.32	708.00	664.00	1416.00	1318.00
Average	15.40	16.82	0.81	0.74	7.81	7.59	793.50	731.73	1586.77	1463.07
Median	15.40	16.60	0.76	0.67	7.82	7.70	746.50	669.00	1493.00	1337.50
Range	0.30	1.90	0.25	0.25	0.26	0.41	235.00	199.00	468.00	408.00
Rank sum	465.00	1365.00	1165.00	665.00	1289.00	541.00	1165.00	665.00	1165.00	665.00
N	30	30	30	30	30	30	30	30	30	30

In = Affluent wastewater to the lagoon; Out = Effluent into Titicaca Lake.

#### 3.1.2 Salinity

The median value of salinity in the affluent (0.76 mg/L) with respect to the median value of the effluent (0.67 mg/L) presented a decrease (Table 3), and these differences are significant (Kruskal Wallis, p<0.05). This indicates evidence of a decrease in ion concentration, as indicated by Chalarca Rodríguez et al. (2007) that a low electrical conductivity is directly related to low salinity, on the other hand, reported by Otero (2011) the values of the affluent are higher than the values of the effluent. This indicates a not very important decrease in salts, due to the sedimentation process in the stabilization lagoon.

#### 3.1.3 pH

The median value of pH (7.82) in the affluent with respect to the median value of pH of the effluent (7.70) decreases (Table 3), and these differences are significant (Kruskal Wallis, p<0.05). Quezada Barahona and Rodríguez Rodríguez (2009) state that the appropriate pH value for different treatment processes and for the existence of biological life can be very restrictive and critical, however, it is generally 6.5 to 8.5. This is also confirmed by D.S.003-2010-MINAN (MINAM, 2010), for the discharges of wastewater to bodies of water. The pH in the effluent turned out to be more alkaline and in the effluent it turned out to be less alkaline, these values possibly could affect the microbial flora of the recipient body, as mentioned by Isasa (2000).

#### 3.1.4 Total dissolved solids

The median value of total dissolved solids in the affluent (746.50 ppm) with respect to the median value of the effluent (669.00 ppm) shows a decrease (Table 3), and these differences are significant (Kruskal Wallis, p<0.05). This variations could be due to some raining during the sampling. Chalarca Rodríguez et al. (2007) indicate that the variation in time and space are important, and likewise the decrease, as a consequence of a removal of the parameter in the stabilization lagoon, The effluent values are lower with respect to the affluent values, this possibly due to an increase in temperature, sedimentation of suspended matter occurred, as indicated by Isasa (2000). Also Lampoglia (2001), mention that the total dissolved solids are directly related to the electrical conductivity.

#### 3.1.5 Electric conductivity

The median value of electrical conductivity in the affluent (1493 μs/cm) with respect to the median value of the effluent (1337.50 μs/cm) showed a decrease (Table 3) and these differences are significant (Kruskal Wallis, p<0.05). This indicates evidence of a decrease in ion concentration, as indicated by Chalarca Rodríguez et al. (2007), electrical conductivity values and salinity values have a direct relationship, Also mentioned by Otero (2011) the values reported in the affluent are higher than the effluent values, which are higher values than those reported by Ruiz Díaz et al. (2016), possibly due to a little sedimentation of total solids in the stabilization lagoon, as indicated by Isasa (2000).

### 3.2 Calculation of the removal efficiency for the parameter of total dissolved solids

**Figure 2:**
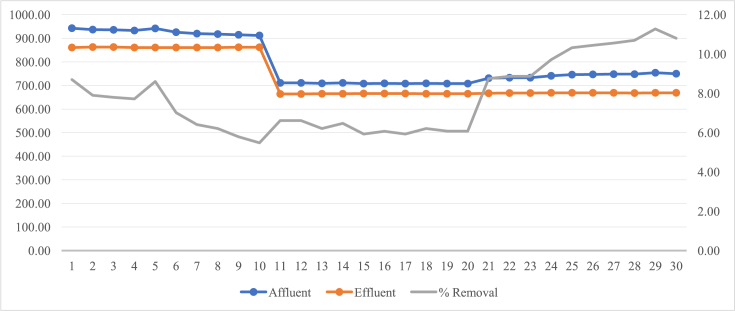
Total dissolved solids data and removal percentage.

Removal it is understood as the ability of the system to remove some of the concentration of pollutants found in wastewater (Romero-Aguilar et al., 2009). Thus, the removal efficiency has been estimated for the parameter of total dissolved solids of each sample (see Figure 2). The parameter presented a 7.80 in average percentage of removal in its journey through the stabilization lagoon (Table 4). Mejía Ruiz (2008) indicates that when there is not a very important removal, it could be an indication of a malfunction of the stabilization lagoon.

**Table 4:** Descriptive statistics of removal percentage.

Descriptive Statistics	*% Removal*
Average	7.80
Median	7.37
Range	5.79
Maximum	11.27
Minimum	5.48
Sum	234.10
N	30

### 3.3 Comparison with the Peruvian standard, on permitted values of concentration of discharges to bodies of water, of wastewater from the effluent of the Espinar stabilization lagoon

A comparison was made of the median effluent pH value (7.70), which is between the maximum permissible limits of pH value (6.5–8.5). The median temperature value (16.60 °C) is lower than the temperature maximum permissible value (<35 °C), there are no values for the parameters of total dissolved solids, electrical conductivity and salinity in the DS-003-2010-MINAM (MINAM, 2010).

## 4 Conclusions

The environmental parameters evaluated in the Espinar stabilization lagoon showed an incremental concentration of the water temperature parameter, and a decreasing concentration in the parameters: pH, total dissolved solids, electrical conductivity and salinity.

After the comparison of values, for the parameters of pH and water temperature, the effluent is below the maximum permissible limits.

The efficiency of 7.80 percent of total dissolved solids removal, presented in the stabilization lagoon is not a very important removal.

Although the result of this analysis has made it possible to demonstrate that the parameters are within the established parameters, it is necessary to implement continuous and timely monitoring, in order to avoid a risk to the environment that surrounds the stabilization lagoon.

## Declarations

### Author contribution statement

Lucio Ticona Carrizales: Conceived and designed the experiments; Performed the experiments; Wrote the paper.

Polan Franbalt Ferró-Gonzales: Conceived and designed the experiments; Analyzed and interpreted the data; Wrote the paper.

Cynthia Milagros Apaza-Panca:Analyzed and interpreted the data; Wrote the paper.

Enrique Gualberto Parillo Sosa, Cristóbal Rufino Yapuchura Saico and Ingrid Rossana Rodríguez Chokewanca: Contributed reagents, materials, analysis tools or data; Wrote the paper.

### Funding statement

This research did not receive any specific grant from funding agencies in the public, commercial, or not-for-profit sectors.

### Data availability statement

The data that has been used is confidential.

### Declarations of interests statement

The authors declare no conflict of interest.

### Additional information

No additional information is available for this paper.
